# Editors' Note

**DOI:** 10.5195/ijt.2023.6563

**Published:** 2023-05-11

**Authors:** Ellen R. Cohn, Jana Cason

## Journal Overview

The International Journal of Telerehabilitation (IJT) is a biannual journal dedicated to advancing telerehabilitation by disseminating peer-reviewed information about current research and practices. IJT is indexed by PubMed and Scopus.

IJT is published via the open journal system (OJS) and sponsored by the University of Pittsburgh's Office of Scholarly Communication and Publishing at the University Library System. This institutional support enables IJT to be subscription free and require no author fees.

## International Readership and Authors

IJT enjoys a diverse international audience due to the expanding global relevance of telemedicine, telehealth, and telerehabilitation. In this Spring 2023 issue, we are pleased to publish work by authors from Brazil, Canada, Italy, Portugal, Ukraine, and the United States.

## Letter to the Editor – Update from Ukraine

We are gratified that a researcher from the Glushkov Institute of Cybernetics of the National Academy of Sciences of Ukraine, Kyrylo Malakhov continues to keep our readership updated on their clinical and research efforts in the form of a Letter to the Editor. The *International Journal of Telerehabilitation* (IJT) was launched fifteen years ago as part of the Department of Education Rehabilitation Research Center on Telerehabilitation. It was inconceivable in 2008 that IJT would be serving as a conduit to report updates from war torn Ukraine. We applaud the resilience of these Ukrainian scientists and clinicians.

